# Antimicrobial Efficacy and Permeability of Various Sealing Materials in Two Different Types of Implant–Abutment Connections

**DOI:** 10.3390/ijms23148031

**Published:** 2022-07-21

**Authors:** Igor Smojver, Roko Bjelica, Marko Vuletić, Dražena Gerbl, Ana Budimir, Dragana Gabrić

**Affiliations:** 1 St. Catherine Specialty Hospital, 10000 Zagreb, Croatia; ismojver@gmail.com; 2 Department of Oral Surgery, School of Dental Medicine, University of Zagreb, 10000 Zagreb, Croatia; rbjelica@sfzg.hr (R.B.); mvuletic@sfzg.hr (M.V.); 3 Department of Dental Medicine, University Hospital Centre Zagreb, 10000 Zagreb, Croatia; 4 Department of Anesthesiology and ICU, University Hospital Centre Zagreb, 10000 Zagreb, Croatia; drazena.gerbl@gmail.com; 5 Department of Clinical and Molecular Microbiology, School of Medicine, University of Zagreb, 10000 Zagreb, Croatia; abudimir@kbc-zagreb.hr; 6 Department of Clinical and Molecular Microbiology, University Hospital Centre Zagreb, 10000 Zagreb, Croatia

**Keywords:** dental implant–abutment design, peri-implantitis, microbial colony count, candida albicans, staphylococcus aureus

## Abstract

The presence of a microgap along an implant–abutment connection (IAC) is considered the main disadvantage of two-piece implant systems. Its existence may lead to mechanical and biological complications. Different IAC designs have been developed to minimise microleakage through the microgap and to increase the stability of prosthodontic abutments. Furthermore, different sealing materials have appeared on the market to seal the gap at the IAC. The purpose of this study was to evaluate the antimicrobial efficacy and permeability of different materials designed to seal the microgap, and their behaviour in conical and straight types of internal IACs. One hundred dental implants with original prosthodontic abutments were divided into two groups of fifty implants according to the type of IAC. Three different sealing materials (GapSeal, Flow.sil, and Oxysafe gel) were applied in the test subgroups. The contamination of implant–abutment assemblies was performed by a joint suspension containing *Candida albicans* and *Staphylococcus aureus*. It was concluded that the IAC type had no significant influence on microleakage regarding microbial infection. No significant difference was found between the various sealing agents. Only one sealing agent (GapSeal) was found to significantly prevent microleakage. A complete hermetic seal was not achieved with any of the sealing agents tested in this study.

## 1. Introduction

Continuous improvements in implant design, surgical protocols, and the development of biologically oriented materials have turned implantology into predictable therapy with a high success rate of 90% [1]. Two-piece implant systems consist of the fixture or the implant itself and a prosthodontic abutment which is connected to the implant [2]. It is precisely the implant–abutment connection (IAC) that is considered the key factor in the long-term stability of the implants and peri-implant tissue health [3]. The main disadvantage of two-piece implant systems is a microgap which persists along the IAC even though the prosthetic abutment is fixed to the implant with the abutment screw [4]. This gap occurs alongside the abutment fixation screw threads and at the bottom of the screw [5]. The value of the microgap varies between 10 and 135 μm, and its existence may lead to biological and mechanical complications [6]. Taking into consideration the average dimensions of bacteria—from 0.2 to 1.5 μm in width and from 1 to 10 μm in length—and the aforementioned microgap values, it is obvious that this space between the prosthodontic abutment and the dental implant acts as a reservoir for microorganisms [4]. Micromovements of the abutment consequently transport microorganisms through the IAC to the interior surface of the implant system and vice versa, which may lead to infections of peri-implant tissues [7]. In the most unfavourable cases, microbial colonisation can lead to peri-implantitis, characterised by rapid peri-implant bone resorption and loss of osseointegration [4]. From a mechanical point of view, a microgap permits micromovements and rotation of the abutment, thus reducing the reverse torque value of the abutment screw, which leads to screw loosening and screw fracture in severe cases [8]. Different IAC designs have been developed to minimise microleakage through the microgap and to increase the stability of prosthodontic abutments. They are primarily divided into internal and external connection types. External IACs were developed first, but internal types are currently more frequently used. Internal IACs, with a connection feature inferior to the coronal surface of the implant, can be divided into straight or clearance-fit, conical, and mixed [3]. Internal connections also exist in various forms, such as hexagon, octagon, cylinder hex, cone screw, and spline [3]. According to the contemporary literature, the internal connection type provides better mechanical and biological outcomes, including a microbial seal, than the external connection type [2]. Due to the possibility of microbial colonisation and leakage at the IAC, materials that are declared to seal the gap at the IAC have appeared on the market [9]. GapSeal (Hager and Werken, Duisburg, Germany) is a sealing material based on a highly viscous silicone matrix with thymol. It has a long-lasting viscosity and can be removed only by ethanol or by mechanical means; therefore, it should provide prolonged protection against auto- and re-infections at the IAC [7]. Thymol has been shown to possess antimicrobial properties such as bacteriostatic activity against most of the Gram-positive and Gram-negative bacteria, and antifungal activity inhibiting *Candida albicans* MTCC 227 biofilm formation [10]. Silicones have been used as sealants and adhesives in a broad variety of fields, dental medicine included. They have a low surface tension and thus are capable of wetting various surfaces. Furthermore, their elastic behaviour enables them to absorb movements without tearing away from the adjacent material or tearing themselves apart. Silicones are also biocompatible and resistant to the dynamic conditions found in the oral cavity [11]. Another sealing material is Oxysafe Gel (Hager and Werken, Duisburg, Germany), which is used in the treatment of periodontitis and peri-implantitis. It contains patented active oxygen technology with antimicrobial activity, but there is a lack of observed data on sealing efficacy [12]. In addition, a sealant based on a polydimethylsiloxane matrix with the addition of thymol is Flow.sil (Bredent GmbH and Co.KG, Senden, Germany). It is declared to ensure reliable sealing through even distribution and thus prevents the harbouring of microorganisms [9].

Since there is no recent literature investigating the efficacy of various sealing materials and comparing their effects in two different types of IACs, the aim of this study was to evaluate, under static conditions, the antimicrobial efficacy and permeability of different materials designed to seal the microgap, and their behaviour in conical and straight types of internal IACs.

The null hypothesis was that there would be no difference in antimicrobial efficacy of the various sealing materials in the two different types of IACs.

## 2. Results

In order to quantitatively describe the samples used in this study, the results were determined based on the frequency of microbial leakage for descriptive purposes. A positive result was signified by the presence of *C. albicans* or *S. aureus*, while the complete absence of these microorganisms gave a negative result.

According to the frequencies of bacterial and fungal microleakage for both straight (Table 1) and conical (Table 2) types of connections, all sealing materials were compared to positive and negative controls regarding *S. aureus* (Table 3) and *C. albicans* (Table 4) infection using Fisher’s exact test. The IAC type had no influence on the internal fit regarding *S. aureus* infection, and there was no statistically significant improvement with the use of different sealing materials in comparison with the control subgroups since the *p*-values were above the level of significance (*p* > 0.05). GapSeal was the only sealing agent that was significantly more efficient compared to the negative control subgroup (*p* = 0.008) (Table 3). The same conclusion could be drawn regarding *C. albicans* infection (Table 4). There was no statistically significant improvement with any sealing material, except GapSeal, compared to the negative control subgroup (*p* = 0.000). The IAC type had no influence on microleakage according to the *p*-values of Fisher’s exact test.

**Figure 1:**
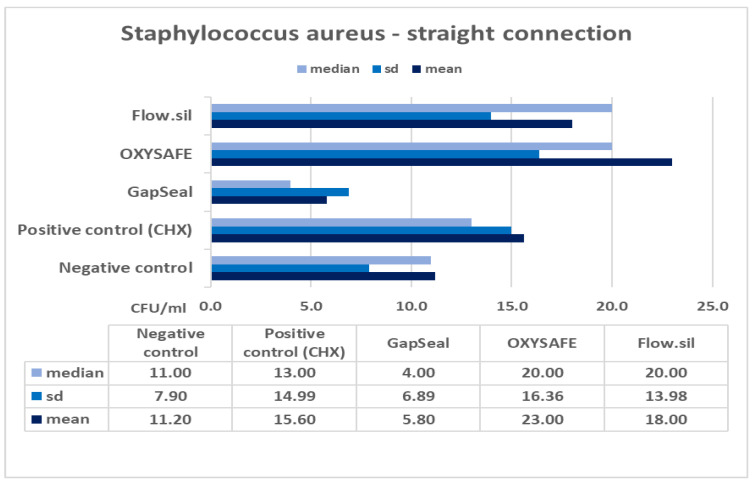
Mean counts of *S. aureus* detected on the internal surface of Zimmer implants and impact of using different sealing materials.

**Figure 2:**
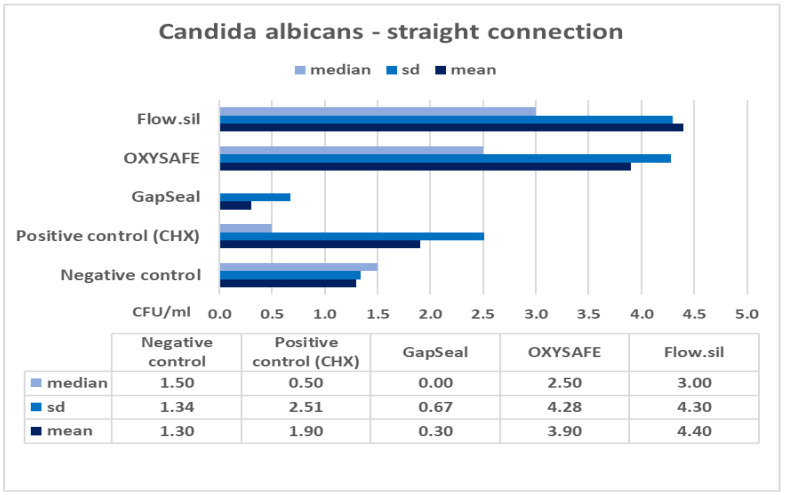
Mean counts of *C. albicans* detected on the internal surface of Zimmer implants and impact of using different sealing materials.

**Figure 3:**
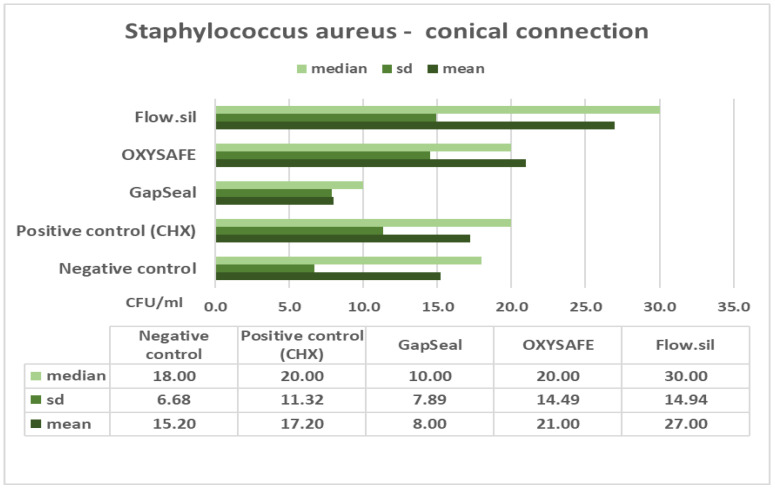
Mean counts of *S. aureus* detected on the internal surface of GC implants and impact of using different sealing materials.

**Figure 4:**
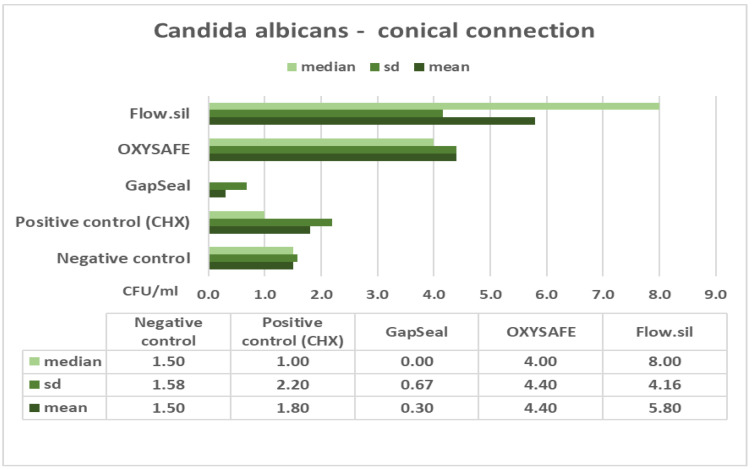
Mean counts of *C. albicans* detected on the internal surface of GC implants and impact of using different sealing materials.

The column charts below (Figure 1, Figure 2, Figure 3 and Figure 4) show the mean counts of *S. aureus* and *C. albicans* detected on the internal surface of the implants depending on the IAC type, and the influence of using different sealing materials on microbial leakage.

## 3. Discussion

In the presented study, two different types of IACs were compared in terms of their sealing efficacy, as well as the antimicrobial efficacy and permeability of different materials designed to seal the microgap. The null hypothesis was accepted regarding the IAC type, with no significant difference in microleakage between straight and conical types of connections (Table 3 and Table 4). The results also show that a complete seal against microbial infection was not achieved at the IAC despite the use of different sealing materials (Figure 1, Figure 2, Figure 3 and Figure 4). Consequently, no significant difference was found between the various sealing agents designed to prevent microleakage. Only one sealing agent (GapSeal) was found to significantly prevent microleakage, especially against *Candida* spp. infection (Table 4). Taking into consideration the pathogenesis of peri-implant diseases, which is largely determined by the constant microbial microflow through the IAC, it is essential to become thoroughly acquainted with different IAC types and the biomechanical features of existing sealing materials.

The bacterial composition of the biofilm is comparable between dental implants and neighbouring teeth, with a vast variety of oral microorganisms accumulating on implant surfaces [13]. Early colonisers are most commonly Gram-positive cocci with the ability to create the preconditions for later colonisation by Gram-negative anaerobic and facultatively anaerobic bacteria [14]. The “red complex” bacteria (*Porphyromonas gingivalis*, *Tannerella forsythia*, and *Treponema denticola*), *Aggregaticabacter actinomycetemcomitans*, and *Fusobacterium nucleatum* are the main pathogens associated with late colonisation of periodontal and peri-implant sites. However, there is strong evidence in numerous studies that the peri-implant microbiome is distinct from the periodontal microbiome, especially in the later stages of the disease [14,15,16]. The microorganisms identified in peri-implantitis that are not commonly detected in periodontitis include *Staphylococcus* spp., *Enterobacter aerogenes, Helicobacter pylori, Pseudomonas* spp., and *Candida* spp. [17]. It can be concluded that peri-implantitis is often associated with opportunistic pathogens. *C. albicans* is one of the many fungal species present in the oral microbiome as a commensal and a major pathogen in oral and systemic candidiasis [18]. It also plays an important role in biofilm arrangement [1]. As a part of colonising bacterial microbiota, *S. aureus* is commonly associated with failed dental implants [17]. According to in vitro studies, it also has a strong affinity to titanium surfaces, which contributes to its role in peri-implant pathology [19]. Due to these data, a decision was made to contaminate the implant–abutment assemblies with a joint microbial suspension containing *C. albicans* and *S. aureus*.

The presented results show that the IAC type had no significant influence on microleakage. It is crucial to identify the most important factors and conditions in which the study was conducted in order to draw a viable conclusion regarding the relationship between the IAC type and microleakage. Tsuruta et al. [20] observed that the amount of microleakage was significantly smaller in Nobel BioCare implants with an internal conical connection than those with an internal parallel connection type, especially after more than 1000 cycles of tensile and compressive loading. These results are not in accordance with those obtained in this study, but the most important factor that needs to be taken into consideration is the dynamic conditions in which the study was conducted. On the other hand, a static in vitro study by Gherlone et al. [21] also demonstrated significantly less microleakage with an internal conical connection in comparison with other internal connections (hexagonal and Morse locking taper). Only 30% of implants with a conical IAC were contaminated with the *Escherichia coli* suspension, whereas the other control internal IAC types were 100% contaminated. In a similar in vitro study to the presented one, Discepoli et al. [22] evaluated microleakage at five different IAC types. No sealing materials were used, but microbial leakage of *S. aureus* was independent of the IAC type. They also concluded that there was a tendency toward a better sealing efficacy against *S. aureus* for internal conical and hexagonal connections. In a recent review by Bittencourt et al. [23], it was concluded that microleakage in the Morse conical connection was lower when compared with the internal and external hexagon connections. According to the literature, perfect sealing at the IAC has not been provided by any implant system, and a complete hermetic seal is not yet achievable [24]. Ardakani et al. [25] observed that microleakage through the IAC occurs in all implant systems, with special emphasis on torquing abutments to 20 N/cm to minimise microbial leakage. This statement is analogously supported by the results of this study in which a complete seal was not achieved either with different IAC types or with the use of sealing materials. It is crucial to point out that microleakage at the IAC is dependent on the torque applied to the system. Larrucea et al. [26] observed no microbial leakage when a 20 and 30 N/cm torque was applied to internal conical connection models infected with *Porphyromonas gingivalis*. However, the contemporary view is that conical and mixed IAC systems behave better regarding the microgap dimensions and consequential amount of leaked microbiota [24,27].

Further analysis of the results showed no statistically significant difference between the various sealing materials used to prevent microleakage at the IAC (Figure 1, Figure 2, Figure 3 and Figure 4). GapSeal was the only material that was significantly more efficient compared to the negative control subgroup. Analysing the descriptive statistics, it is clear that the presence of media at the IAC reduces leakage, especially with the use of GapSeal (Table 1 and Table 2). The improvements made by the sealing agents can be explained either by having antimicrobial properties or a pure mechanical sealing ability. GapSeal has been found to reduce microleakage at the IAC after dynamic loading in different implant systems with an internal conical connection [28]. Seloto et al. [29] concluded that Loctite 2400 sealing gel contributed to the sealing efficacy of the IAC by decreasing vertical misfit values. It is worth mentioning that the implants used in that study had an external hexagonal connection, and thus the results cannot be directly compared with those of the present study. In a recent study by Smojver et al. [9], it was observed that the presence of GapSeal material significantly reduced microleakage at the IAC. These results are in accordance with the results obtained by Nayak et al. [30], who saw the least growth of *Enterococci* when the GapSeal sealing agent was used. The above-stated results accord well with those of the presented study and provide solid proof of GapSeal’s usefulness. On the other hand, Mohammadi et al. [31] found that Atridox significantly delayed bacterial microleakage when compared to other materials, including GapSeal. In addition, it was concluded that a complete hermetic seal against microbial infection is not achievable despite the possible reduction in microbiota found at the IAC.

Finally, since there is no literature investigating the efficacy of various sealing materials and comparing their effects in two different types of IACs, valuable results were attained. The sample size of 100 dental implants was sufficient to analyse the differences between the two types of IACs and the effects of various sealing materials on microleakage. However, due to the presence of three different sealing agents, a larger sample size would be better. It is of great importance to re-emphasise that this was an in vitro study and further clinical research is necessary to evaluate the obtained results.

## 4. Materials and Methods

### 4.1. Study Design

This in vitro pilot study was conducted according to the guidelines of the Declaration of Helsinki and was approved by the Ethics Committee of the School of Dental Medicine, University of Zagreb (protocol code: 05-PA-30-XII-12/2019 73 on 5 December 2019). Microbiological preparation, sampling, and processing of samples were performed at the laboratory of the Department of Clinical and Molecular Microbiology, University Hospital Centre Zagreb. The methodology was developed based on the protocol used in the recent pilot study by Smojver et al. [32], which has been tested repeatedly for in vitro static conditions.

**Figure 5:**
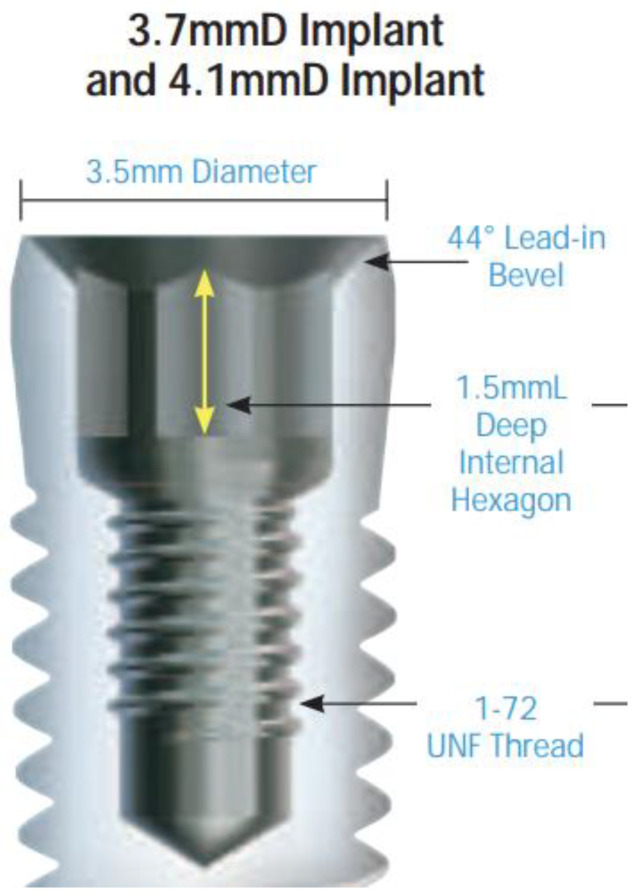
Zimmer Tapered Screw-Vent implant with the straight type of internal IAC. Reprinted with permission from the manufacturer.

**Figure 6:**
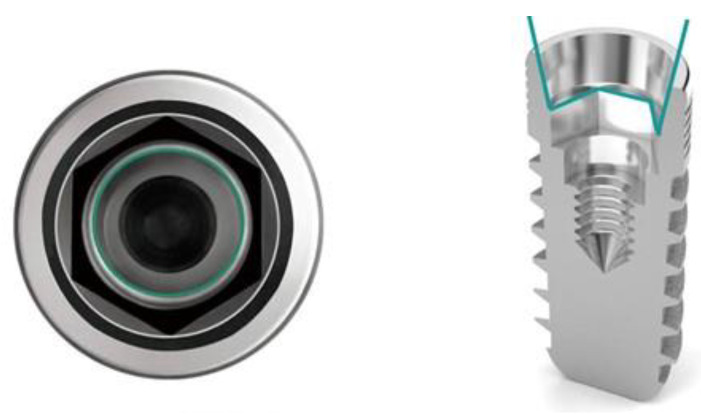
GC Aadva Standard implant with the conical type of internal IAC. Reprinted with permission from the manufacturer.

One hundred dental implants with original prosthodontic abutments were evaluated in this study and divided into two main groups of fifty implants according to the type of IAC. The implants used in this study were Zimmer Tapered Screw-Vent implants (Zimmer Biomet Dental, Palm Beach Gardens, FL, USA) with a straight connection (Figure 5) and GC Aadva Standard implants (GCTech.Europe GmbH, Breckerfeld, Germany) with a conical connection (Figure 6).

Group 1 comprised 50 GC Aadva Standard implants (GCTech.Europe GmbH, Breckerfeld, Germany) of 4.0 mm diameter, connected to the original prosthodontic abutments.

Group 2 comprised 50 Zimmer Tapered Screw-Vent implants (Zimmer Biomet Dental, Palm Beach Gardens, FL, USA) of 4.1 mm diameter, also connected to the original prosthodontic abutments.

In each of the main groups (1 and 2), three subgroups of 10 implants were formed for different sealing materials, as follows:

1. GapSeal gel (Hager and Werken, Duisburg, Germany);
2. Oxysafe gel (Hager and Werken, Duisburg, Germany);
3. Flow.sil (Bredent GmbH and Co.KG, Senden, Germany).

**Figure 7:**
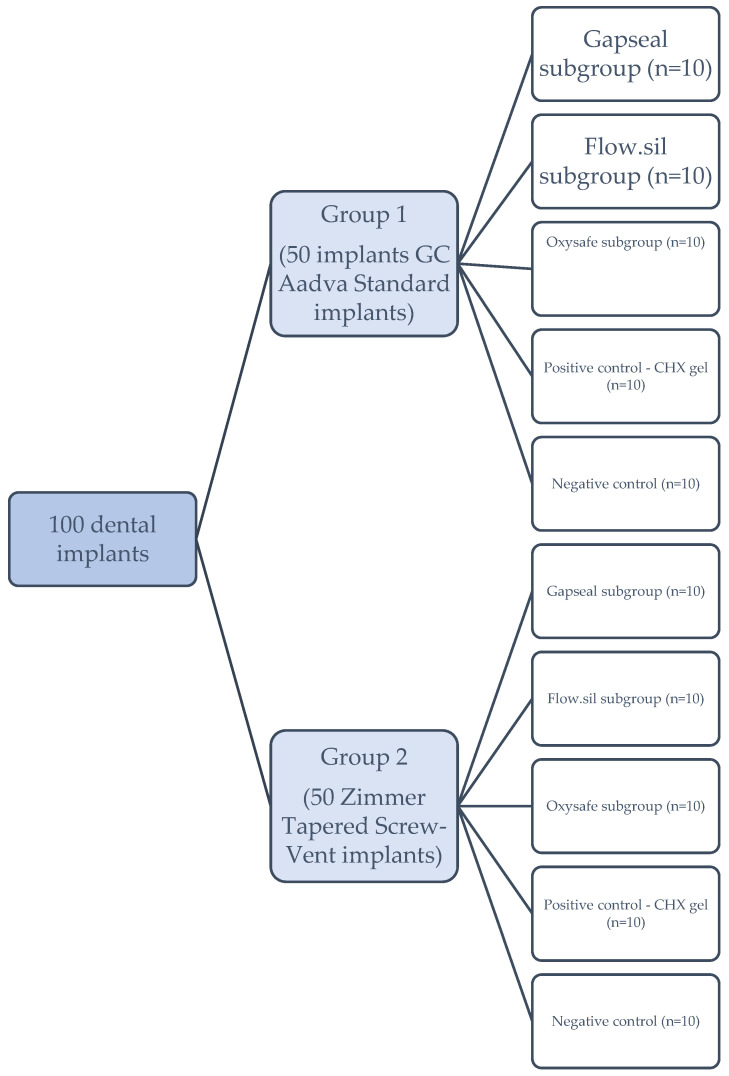
Study design and division of the implants into groups.

One positive control subgroup of 10 implants with chlorhexidine gel (Curasept ADS 350 gel, Curaden International AG, Kriens, Switzerland) and one negative control subgroup without sealant (10 implants) were also formed in each of the main groups (Figure 7).

### 4.2. Implant–Abutment Assembly Preparation

**Figure 8:**
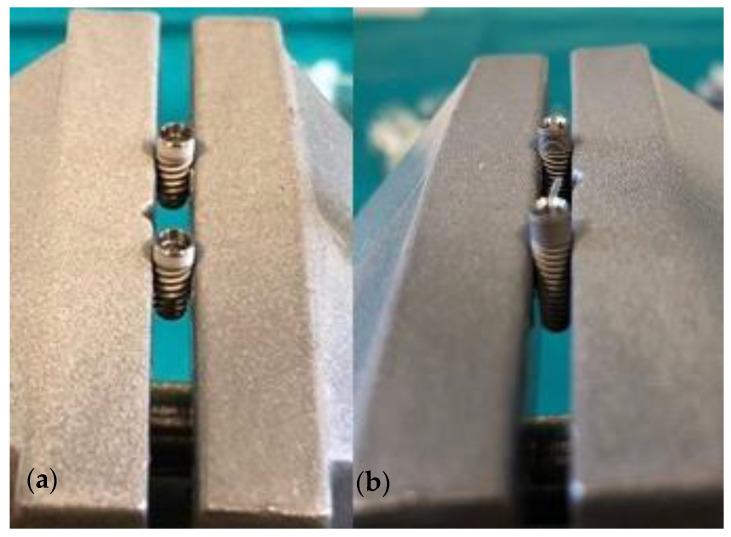
(**a**) GC implants (conical IAC type) in a sterile clamp; (**b**) Zimmer implants (straight IAC type) in a sterile clamp.

Every dental implant was removed from its commercial package and placed in a sterile stainless-steel clamp using sterile forceps (Henry Schein, Melville, NY, USA). All the instruments used in this study were sterilised in Euroklav 23 VS+ (Melag, Berlin, Germany). Implants were placed into the clamp in a strictly vertical position (Figure 8) which enabled a firm rotational movement during the tightening of the prosthodontic abutments.

**Figure 9:**
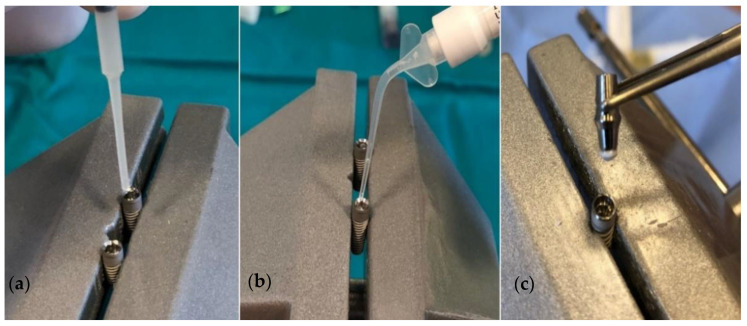
Application of the sealing materials. (**a**) GapSeal; (**b**) Oxysafe; (**c**) Flow.sil.

Sterile brain heart infusion (BHI) broth (0.3 µL) (calf brains (12.5 g/L), beef heart infusion solids (5.0 g/L), proteose peptone (10.0 g/L), D-glucose (2.0 g/L), sodium chloride (5.0 g/L), and disodium hydrogen phosphate (2.5 g/L) at a pH 7.4 ± 0.2 and 25 °C) was added to the internal surface of the implants using a sterile micropipette (Merck KGaA, Darmstadt, Germany) to serve as a non-selective nutrient medium in case of microbial penetration. The sealing material was then applied to the internal surface of the implants in the test subgroups (GapSeal, Oxysafe, or Flow.sil) depending on the subgroup (Figure 9). Application of the sealing materials was performed strictly according to the recommendations from the manufacturers. Chlorhexidine gel is considered an effective antimicrobial agent used in different fields of dentistry and therefore was applied in the positive control subgroup [33]. The negative control subgroup did not receive any material.

**Figure 10:**
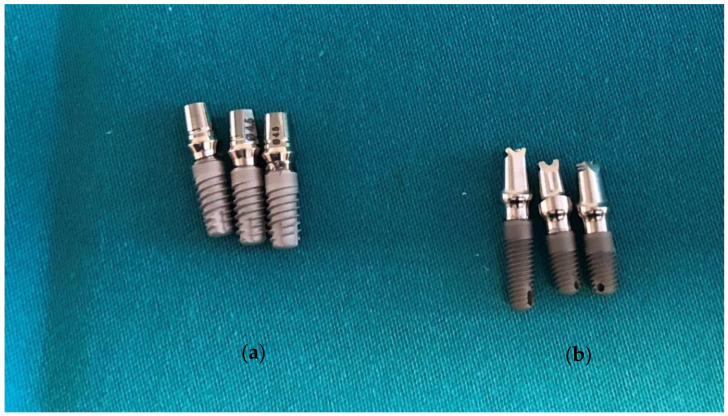
(**a**) GC implants with original prosthodontic abutments; (**b**) Zimmer implants with original prosthodontic abutments.

Original prosthodontic abutments were removed from their commercial packaging under sterile conditions and placed into the implants (Figure 10) using the values recommended by the manufacturers (30 N/cm for Zimmer Tapered Screw-Vent and 20 N/cm for GC Aadva Standard implants).

### 4.3. Contamination Procedure

*Candida albicans* and *Staphylococcus aureus* strains were isolated from a clinical sample at the Clinical Hospital Centre Zagreb. Separated isolation of the fungal and bacterial strains was performed in Columbia Agar for 72 h. Suspensions were prepared using thioglycolate broth, following their mixture into a single, joint suspension. The suspension density was set to 600 nm using an optical densitometer (Densimat, Biomerieux, Marcyl’Etoile, France). This value is equivalent to 1 × 10^8^ colony-forming units per millilitre (CFU/mL).

Contamination of implant–abutment assemblies was performed by immersing them in 300 μL of joint microbial suspension (containing *C. albicans* and *S. aureus* at a density of 0.5 McFarland) for 14 days under aerobic conditions. The incubation temperature was set at 35 °C. The microbial suspension covered the neck of the implant and cervical part of the abutment, but the access hole for the abutment screw remained above the suspension level to eliminate the possible impact of penetration of the suspension along the fixation screw and thus false positive contamination.

After the incubation period, the implant–abutment assemblies were removed from Eppendorf tubes using sterile forceps. External contamination was prevented by their immersion in 70% ethanol for 2 min following drying of the samples with a sterile gauze and placement in a sterile stainless-steel clamp. Then, disassembly in a strictly vertical position was performed. The internal surface of the implants was sampled using paper points (Absorbent points, DENTSPLY Maillefer, Tulsa, OK, USA) which were placed into 0.5 mL of sterile phosphate-buffered saline (PBS) in Eppendorf tubes. The tubes containing paper points and PBS were inserted into a vortex mixer (Corning^®^ LSE™ vortex mixer, Corning, NY, USA) for 60 s to suspend fungal and bacterial cells.

**Figure 11:**
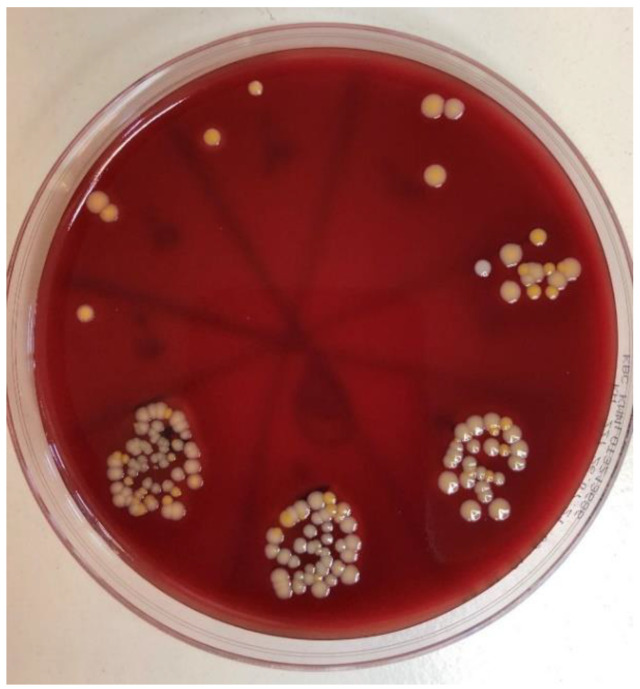
The 5% blood agar with colonies of *S. aureus* and *C.albicans* ready for CFU/mL analysis.

Samples from the Eppendorf tube contents were transferred to 5% blood agar with an incubation period of 48 h and a temperature of 37 °C. Identification and quantification of the resulting colonies (Figure 11) were verified using a MALDI Biotyper (Bruker Daltonics, Hamburg, Germany). Microbial contamination (CFU/mL) was counted for each sample, and the obtained results underwent further statistical analysis.

### 4.4. Statistical Analysis

Fisher’s exact test was used to perform statistical analysis using MedCalc software version 20.014 (MedCalc Software Ltd., Ostend, Belgium). The traditional level of statistical significance was set at *p* < 0.05.

## 5. Conclusions

Based on the results of this study, it can be concluded that the IAC type has no significant influence on microleakage regarding microbial infection. Additionally, GapSeal significantly reduces microleakage, especially against *Candida* spp. infection. Despite the plausible benefits of GapSeal application, a complete hermetic seal was not achieved with any of the sealing agents used in this study. Further clinical research with longer follow-up periods should be conducted to evaluate the effects of using different sealing materials at various IAC types.

## Figures and Tables

**Table 1:** Frequencies of bacterial and fungal microleakage (Zimmer Tapered Screw-Vent implants).

	Flow.sil	OXYSAFE	GapSeal	Positive Control (CHX)	Negative Control
*S.aureus*	80.00%	80.00%	50.00%	70.00%	80.00%
*C. albicans*	70.00%	60.00%	20.00%	50.00%	60.00%

**Table 2:** Frequencies of bacterial and fungal microleakage (GC Aadva Standard implants).

	Flow.sil	OXYSAFE	GapSeal	Positive Control (CHX)	Negative Control
*S.aureus*	90.00%	90.00%	60.00%	80.00%	90.00%
*C. albicans*	70.00%	60.00%	20.00%	50.00%	60.00%

**Table 3:** Comparison of Fisher’s exact test values for microleakage between sealing materials and control subgroups regarding *S. aureus* infection.

Connection Type	Subgroup	Fisher Exact Test (*p* Values)		
		Flow.sil	Oxysafe	GapSeal
**straight and conical**	positive (CHX)	H0 not rejected (0.465)	H0 not rejected (0.465)	H0 not rejected (0.320)
	negative (no seal)	H0 not rejected (0.356)	H0 not rejected (0.605)	H0 rejected (0.008) *
**straight**	positive (CHX)	H0 not rejected (1.000)	H0 not rejected (1.000)	H0 not rejected (0.650)
	negative (no seal)	H0 not rejected (1.000)	H0 not rejected (1.000)	H0 not rejected (0.350)
**conical**	positive (CHX)	H0 not rejected (0.605)	H0 not rejected (0.605)	H0 not rejected (0.628)
	negative (no seal)	H0 not rejected (1.000)	H0 not rejected (1.000)	H0 not rejected (0.303)

* Statistically significant (*p* < 0.05).

**Table 4:** Comparison of Fisher’s exact test values for microleakage between sealing materials and control subgroups regarding *C. albicans* infection.

Connection Type	Subgroup	Fisher Exact Test (*p* Values)		
		Flow.sil	Oxysafe	GapSeal
**straight and conical**	positive (CHX)	H0 not rejected (0.333)	H0 not rejected (0.751)	H0 not rejected (0.096)
	negative (no seal)	H0 not rejected (0.235)	H0 not rejected (0.065)	H0 rejected (0.000) *
**straight**	positive (CHX)	H0 not rejected (0.650)	H0 not rejected (1.000)	H0 not rejected (0.350)
	negative (no seal)	H0 not rejected (1.000)	H0 not rejected (1.000)	H0 not rejected (0.170)
**conical**	positive (CHX)	H0 not rejected (0.650)	H0 not rejected (1.000)	H0 not rejected (0.350)
	negative (no seal)	H0 not rejected (1.000)	H0 not rejected (1.000)	H0 not rejected (0.170)

* Statistically significant (*p* < 0.05).

## Data Availability

The data presented in this study are available on request from the corresponding author.
